# Identifying the Important HIV-1 Recombination Breakpoints

**DOI:** 10.1371/journal.pcbi.1000178

**Published:** 2008-09-12

**Authors:** John Archer, John W. Pinney, Jun Fan, Etienne Simon-Loriere, Eric J. Arts, Matteo Negroni, David L. Robertson

**Affiliations:** 1 Faculty of Life Sciences, University of Manchester, Manchester, United Kingdom; 2 Architecture et Réactivité des ARN, Université Louis Pasteur de Strasbourg, CNRS, IBMC, Strasbourg, France; 3 Case Western Reserve University, Cleveland, Ohio, United States of America; 4 Institut Pasteur, Paris, France; ETH Zürich, Switzerland

## Abstract

Recombinant HIV-1 genomes contribute significantly to the diversity of variants within the HIV/AIDS pandemic. It is assumed that some of these mosaic genomes may have novel properties that have led to their prevalence, particularly in the case of the circulating recombinant forms (CRFs). In regions of the HIV-1 genome where recombination has a tendency to convey a selective advantage to the virus, we predict that the distribution of breakpoints—the identifiable boundaries that delimit the mosaic structure—will deviate from the underlying null distribution. To test this hypothesis, we generate a probabilistic model of HIV-1 copy-choice recombination and compare the predicted breakpoint distribution to the distribution from the HIV/AIDS pandemic. Across much of the HIV-1 genome, we find that the observed frequencies of inter-subtype recombination are predicted accurately by our model. This observation strongly indicates that in these regions a probabilistic model, dependent on local sequence identity, is sufficient to explain breakpoint locations. In regions where there is a significant over- (either side of the *env* gene) or under- (short regions within *gag*, *pol*, and most of *env*) representation of breakpoints, we infer natural selection to be influencing the recombination pattern. The paucity of recombination breakpoints within most of the envelope gene indicates that recombinants generated in this region are less likely to be successful. The breakpoints at a higher frequency than predicted by our model are approximately at either side of *env*, indicating increased selection for these recombinants as a consequence of this region, or at least part of it, having a tendency to be recombined as an entire unit. Our findings thus provide the first clear indication of the existence of a specific portion of the genome that deviates from a probabilistic null model for recombination. This suggests that, despite the wide diversity of recombinant forms seen in the viral population, only a minority of recombination events appear to be of significance to the evolution of HIV-1.

## Author Summary

Multiple variants of HIV can infect the same cell, and because each viral particle contains two copies of the viral genomic RNA, RNAs from different viruses can occasionally be incorporated together within a viral particle. When this virus subsequently infects another cell, genetic exchange (recombination) may occur between these two divergent copies of genomic RNA as a result of a switch between the RNA molecules while they are copied into DNA. This process is very important to understand as it contributes to the generation of new HIV variants. In this study, we have analysed a set of recombinant HIV genomes generated in the laboratory to construct a probabilistic model of the propensity for the switch to take place in specific regions of the genome, dependent on the local similarity of the parental viral sequences. This model allows us to predict the locations where recombination should occur more frequently. By comparing these predictions to the patterns of recombination observed in the HIV-1 pandemic, we identify the genomic regions in which recombination has been more important, in that it has provided an evolutionary advantage to the virus.

## Introduction

The causative agent of AIDS, HIV, exhibits a high rate of evolution as a direct result of the error-prone nature of reverse transcriptase and its tendency to switch between RNA templates [1],[2]. These mutational events, combined with high levels of viral turnover [3],[4]—and diversifying selection due to the action of the immune response [5]–[7]—generate the extreme diversity observed within infected individuals and in the pandemic as a whole [8]. As a consequence of the epidemiological history of the HIV-1 pandemic, specifically the key role of founder effects [9],[10], the global diversity is partitioned into distinct phylogenetic clusters, termed subtypes [11]. If dual infection or superinfection with viruses from different subtypes occurs, recombination can generate an inter-subtype recombinant [12]–[15]. When an inter-subtype recombinant is transmitted between multiple individuals, i.e., has the potential to be of epidemiological significance, it is termed a Circulating Recombinant Form (CRF) [11]. As with the subtypes, these form distinct clusters in phylogenetic trees and some (CRF01 and 02 in particular) contribute disproportionately to the pandemic, as do certain subtypes (particularly C). Superinfection and thus recombination also occurs between viruses from the same subtype or CRF [15]–[18], but these are harder to detect by phylogenetic analyses due to the lack of phylogenetic substructure within subtypes and CRFs.

It is widely assumed that the HIV recombinants have novel properties that led directly to their prevalence, particularly in the case of the CRFs [19]. As a result, enormous effort is expended on characterising CRFs both geographically and in terms of the precise location of the strand-switches, the recombination breakpoints that delimit their mosaic structure. We hypothesise that only a subset of recombination breakpoints will convey any selective advantage. Given the already noted propensity of reverse transcriptase to switch RNA templates, the null hypothesis is that the majority of recombination breakpoints are selectively neutral with limited biological significance, i.e., recombination patterns are adequately explained by strand-switching and have limited impact on viral fitness and evolution.

Factors known to promote strand-switching, and hence recombination, include sequence identity [20],[21] and additional features of the RNA such as homopolymeric runs [22] and secondary RNA structure [23],[24]. Here we are particularly interested in the role of sequence identity and the propensity for recombination to occur. It has been established that high sequence identity between the two RNAs [1],[2], and particularly local sequence identity, is important for efficient strand-switching [21]. This is because strand-switching results from the transfer of the nascent DNA from one RNA (the donor) onto the other (the acceptor) [25]. After this transfer, synthesis must be resumed on the acceptor RNA. Discordant residues between donor and acceptor RNAs result in mismatches in the heteroduplex formed by the nascent DNA and the acceptor RNA, and destabilise it. An unstable heteroduplex near the 3′-OH of the nascent DNA does not constitute a suitable structure for priming reverse transcription on the acceptor RNA and, by making resumption of reverse transcription on the acceptor RNA less efficient, decreases the probability of successful template switching.

**Figure 1:**
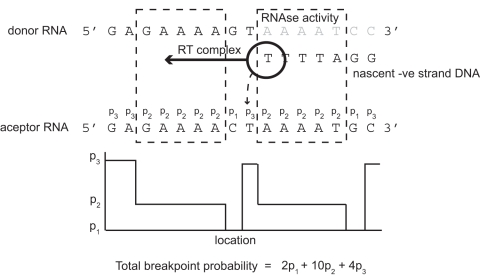
Model of HIV copy-choice recombination. The reverse transcriptase (RT) complex is shown moving from the 3′ end of the donor RNA to the 5′ end. The RNAse activity of RT is indicated by the light grey nucleotides on the donor RNA. The nascent negative DNA strand can be observed to the right of the RT complex. A potential strand-transfer event by RT is indicated by the dashed arrow. The dashed boxes indicate windows of decreased probability of crossover that have been anchored to the 5′ side of each mismatch. The probability of a crossover occurring at each base on the acceptor strand is indicated by *p*_1_, *p*_2_, or *p*_3_ as described in Methods. The plot along the bottom is a representation of each of the probability values across the sequence. For this stretch of 16 nucleotides, the total probability of a crossover occurring is given by the equation shown.

To test our hypothesis regarding the importance of breakpoints, we generate a probabilistic model for the copy-choice recombination process (Figure 1). This takes into account the local sequence identity between the co-packaged RNA genomes and produces an expected null distribution of breakpoints across two parental sequences. The model is based on an analysis of 162 inter-subtype recombinant forms generated in the laboratory in an experimental setting where no selection is applied to the recombinant products [21]. As we have the exact parental sequences, the location of the breakpoints can be determined accurately, i.e., to the nearest mismatch either side of the identical region in which the switch has occurred. This permits a detailed understanding of the influence of sequence identity on template switching. We use this model to generate a null distribution for the observed recombinant breakpoints from the global HIV-1/AIDS pandemic. Regions that deviate from this expected distribution we infer to include the breakpoints that are of greater importance as a consequence of the mosaic structures they have generated. Our results strongly indicate that it is approximately on either side of the envelope gene, or at least gp120, that most of the recombination of significance is occurring, possibly as a result of this region's major involvement in immune evasion.

## Results

**Figure 2:**
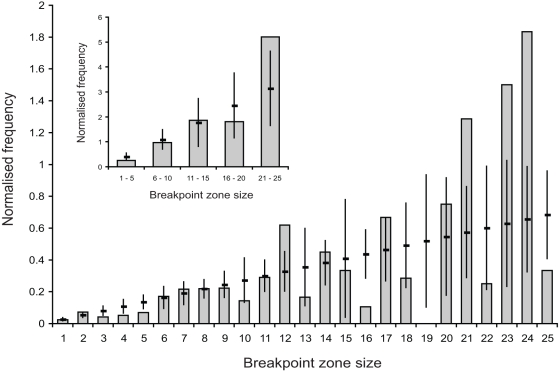
The significance of local sequence identity to recombination. The main plot displays the normalized distribution of in vitro breakpoints falling within zones ranging from size 1 to 25 (vertical grey bars); see Baird et al. [21] for further details. The horizontal lines indicate the expected random distribution of breakpoints for the zones. The inset plot shows the normalised frequency of both the in vitro breakpoints and randomly generated breakpoints for zones up to size 25 (arranged in groups of five). On the main plot, error bars on the random distributions (vertical lines) represent one standard error to include 68.3% of the distribution. On the inset, the error bars on the random distributions represent 1.96×standard error to include 95% of the distribution.

For the 162 inter-subtype recombinant forms generated in vitro [21], we observed that significantly fewer breakpoints were located within five nucleotides of a mismatch between the aligned parental strains than expected under a random distribution of breakpoint locations (*P*<0.05; Figure 2, inset). Note, although the number of breakpoints in zones 16, 19, and 25 are also relatively low (Figure 2) this is most probably due to a lack of data as in the pooled data they are not significantly lower than random (*P*>0.05; Figure 2, inset).

Based on this observation, we developed a sequence identity-dependent probabilistic model (Figure 1) to describe the expected locations of breakpoints without the influence of natural selection. Sequence identity is accounted for by not permitting breakpoints to occur directly on mismatches and by reducing the probability of a breakpoint occurring within a window of size five nucleotides anchored to the 5′ end of each mismatch (see Methods). Windows can potentially overlap within regions of low sequence identity, i.e., regions in which many mismatches are present. The result is that the probability of breakpoints occurring across such regions will be uniformly decreased.

**Figure 3:**
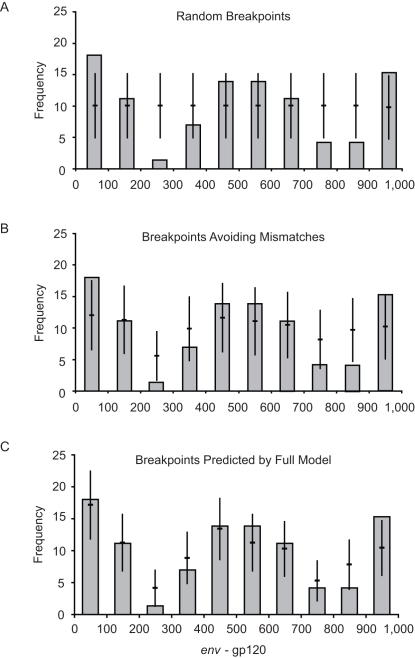
Testing of the recombination models. The probabilistic distribution of breakpoints as predicted by each model (horizontal lines) compared to the in vitro distribution of breakpoints (vertical grey bars). The three panels correspond to predicted breakpoint distributions that ignored sequence identity (A), prohibited a breakpoint on a mismatch (B) and the full model (C); see Methods for further details. The error bars (vertical lines) on the predicted values represent 1.96×standard error to include 95% of the distribution.

The model accurately predicts the breakpoint distribution in the experimental data across the envelope gene, with 9/10 of the 100-nucleotide regions falling within 1.96 standard errors of the predicted values (Figure 3C; *P*>0.05, Chi^2^ test). Simpler models that either (i) used a completely random distribution (ignoring sequence identity) or (ii) prohibited breakpoints to occur directly on a mismatch but omitted the reduced-probability window, produced expected distributions that were significantly different to the experimental distribution of breakpoints (Figure 3A and 3B; *P*<0.001 and *P*<0.01 respectively, Chi^2^ test). When the predictions generated by the full model were compared to those from the simpler “mismatch only” model using an *F*-test, a significant increase in the accuracy of the predications in relation to the experimental data was still observed (*P*<0.05). This indicates that the full model provides a reliable prediction for the null distribution of HIV-1 recombination breakpoints expected in the absence of natural selection.

**Figure 4:**
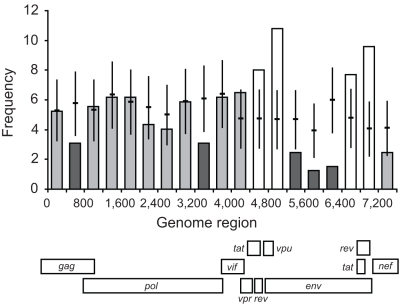
Implementation of the recombination model. Comparison of model-predicted breakpoints (horizontal lines) with breakpoint locations from HIV-1 recombinants (vertical bars) [26] from the HIV Sequence Database. White bars indicate where the number of breakpoints for the global data is significantly higher than the prediction for the region, light grey bars indicate where the global data falls within the prediction, while dark grey indicates where the global data is significantly lower than the model prediction. The error bars on the model-predicted values represent 1.645×standard error to include 90% of the distribution. The normalised frequency data (*y*-axis) have been divided into bins of size 400 nucleotides (*x*-axis). Below the *x*-axis, the various genomic regions of the HIV-1 genome are displayed. Note, positioning of genes is relative to a gap-stripped sequence alignment.

Using this model, the pattern of HIV-1 inter-subtype breakpoints across the whole viral genome was predicted based on representative parental subtypes from the Los Alamos HIV Sequence Database. The predicted distributions were compared to the distributions of inter-subtype breakpoints derived from complete genome HIV-1 recombinants (Figure 4). Across much of the HIV-1 genome, we find that the observed inter-subtype breakpoint frequencies fall within a 90% confidence interval (1.645 standard errors) of those predicted by the model. This observation strongly indicates that within these regions an entirely mechanistic process—mainly due to the local similarity of the parental sequences—is sufficient to explain breakpoint locations. Regions that significantly deviate from these predictions can be identified where there is a significant over- (approximately either side of the *env* gene) or under- (short regions within *gag*, *pol*, and most of *env*) representation of breakpoints (Figure 4). We infer the former to be due to breakpoints that have a tendency to be of greater importance; that is significantly more recombination events are observed in these regions than predicted by the model. Note, this definition of important recombination events does not preclude the occurrence of significant recombination events elsewhere in the genome, just that such events having any selective significance—as a consequence of the recombinants they generate—will be relatively rare.

## Discussion

Our analysis confirms that local sequence identity between the genomic RNAs has a major influence on the probability of a strand-transfer event [19], with significantly fewer breakpoints than expected by chance found within zones of five nucleotides or less of a residue discordant between the two genomic RNAs (Figure 2, inset). This tendency for a reduced probability of template switching permits us to accurately model the expected distribution of recombination breakpoints for any given pair of parental sequences (Figure 3). Applying this model to recombinant sequences from the HIV-1 pandemic (Figure 4) we find that (i) the distribution of recombinant breakpoints across HIV-1's genome is, for the most part, adequately accounted for by a simple model of recombination dependent on sequence identity, and (ii) genomic regions can be identified where there are either more or fewer breakpoints than predicted. This validates our hypothesis that only a subset of recombination events should be considered important, i.e., to convey any selective advantage to the virus.

We consider these important recombination breakpoints to be those over-represented with respect to the distribution predicted by our model. The most evident case is constituted by breakpoints at either side of *env*, which indicates a tendency for the shuttling of the entire envelope gene, or at least the coding region for extra-cellular gp120 (Figure 4). Whether this is a result of coincidental or sequential recombination events, it indicates that selection is frequently promoting *env*'s transfer from one genetic background into another. This tendency for most of *env* to be recombined effectively as an integral unit must be directly related to the envelope protein's functional significance in relation to viral fitness determinants, and in particular its propensity to be subject to high levels of positive selection as a direct result of the action of the immune response on HIV's envelope gene [6],[7],[26],[27].

The paucity of recombination breakpoints within the envelope gene itself (but also in parts of *gag* and *pol*) indicates that recombinants with breakpoints in these regions have a tendency to be selected against. This is presumably due to constraints arising from inter-dependencies within gene and genomic regions [28] as a consequence of the maintenance of protein structural and functional integrity in the context of high viral diversity. Such inter-dependencies are probably related to co-variation of sites [29], for example, purifying selection acting to maintain protein folds [30] or, in *env*, to maintain glycosylation patterns [31] critical for the evasion of neutralising antibodies [32]. Recombination patterns thus have the potential to provide insight into key dependencies between intra- and inter-genic regions.

Our results emphasise that detailed mapping of individual HIV-1 recombinant structures should be considered in the context of a probabilistic expectation generated by the process of template switching during reverse transcription. This underlines the importance of determining which recombination breakpoints are the most important in the maintenance of a persistent infection. Individual recombination breakpoints, analogous to point mutations, will have varying consequences for viral persistence in infected individuals and populations. Further fine-scale mapping of recombination distributions is required to understand more precisely the significance of recombination breakpoints, for example, related to escape from immune control [18] and epistatic interactions [33],[34]. We would also expect to find recombination hotspots in data associated with drug resistance [35]–[37]. In conclusion, our findings provide a clear indication that the majority of recombinant breakpoints detected in the HIV-1 pandemic provide limited selective advantage, with the exception being specific genomic regions in which recombination events have a higher probability of being important to viral evolution.

## Methods

### In Vitro Breakpoint Distributions

The frequencies of recombination breakpoints occurring at different locations across 162 in vitro recombinant sequences were obtained in a previous study [21]. The frequency of breakpoints falling within a breakpoint zone (the region of identity between two mismatches) of a particular size was calculated. The frequency for each zone size was normalised by dividing the number of observed breakpoint occurrences by the total number of potential breakpoint zones of that size. We only considered zones up to size 25 nucleotides because for larger zone sizes the limited number of recombinant sequences meant that data were sparse.

### Probabilistic Model for Breakpoint Distributions

Three independent methods were used to generate predicted recombination breakpoint frequencies: (i) random breakpoint prediction, (ii) breakpoint prediction based on mismatch locations only, and (iii) breakpoint prediction using our full model.

(i) The probability, *p_b_*, of creating a random breakpoint on any site within the parental alignment is given by
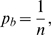
(1)where *n* is the length of the alignment.

(ii) To take sequence identity into account, the probability of a breakpoint being located on a mismatch is reduced to zero. The probability of creating a breakpoint on any site that is not a mismatch, *p_b_′*, becomes
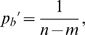
(2)where *m* is the number of mismatches in the alignment.

(iii) In the full model (Figure 1) there are three different categories of site. These are: (a) sites located on mismatches, (b) sites located within windows of size five nucleotides downstream of a mismatch, and (c) sites located neither on a mismatch nor within a window. At each type of site, the probability of a breakpoint occurring is given by *p*_1_, *p*_2_, or *p*_3_, respectively. Across the full alignment, the sum of probabilities over all sites is 1. The model can therefore be summarized as
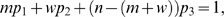
(3)where *w* is the number of nucleotides falling within a window. Since breakpoints should not occur where there is a mismatch, *p*_1_ is set to zero. We further define the ratio
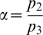
(4)to represent the factor by which the probability of recombination is reduced within a window. From Equation 3, the model parameters can therefore be expressed as
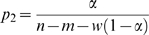
(5)and
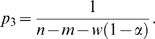
(6)


To estimate the value of *α*, a line of best fit was drawn through the normalised in vitro breakpoint frequency data for zones of size five or less. A second line of best fit was drawn through the data for zones of greater than size five. Since the gradient of such a line corresponds to the average recombination frequency associated with a single nucleotide falling in a specific category (window/non-window), the ratio of the gradients can be used to give a value of *α* = 0.37.

The model has been implemented in Java and the source code is available from the authors on request.

### Global Breakpoint Distribution

Unique breakpoints from 80 inter-subtype recombinants from the Los Alamos HIV Sequence Database [26] were used to obtain the distribution of breakpoints across the full length of the HIV-1 genome in 400 nucleotide bins. This in vivo or “global” data included 324 breakpoints, after the exclusion of 30 breakpoints that bordered unclassified regions. A bin size of 400 was chosen due to the sparsity of the data. This size is sufficiently small to capture the large-scale variation in breakpoint frequencies between different regions of the genome; a finer scale analysis will require more data.

To predict the distribution of inter-subtype recombination across HIV's genome, the probabilities of breakpoints occurring at individual sites were calculated from Equations 5 and 6. The probability of a breakpoint occurring on a site where there was a mismatch between the two parental sequences was set to zero. The parental strains used to represent the group M subtypes included within the global recombinants were: AF069670 (subtype A), K03455 (subtype B), AF067155 (subtype C), U88824 (subtype D), AF005494 (subtype F), AF061641 (subtype G), AF190128 (subtype H), AF082394 (subtype J), and AJ249235 (subtype K). Sites were grouped into 400 nucleotide bins and probabilities were summed across all parental pairs and weighted according to the number of breakpoints that were observed for the same parental pair. The numbers of inter-subtype recombinants modelled were: AB, 2; AC, 50; AD, 83; AG, 35; AJ, 8; AK, 4; BC, 14; BF, 66; BG, 2; CD, 19; CG, 5; DF, 7; FK, 7; GH, 5; GJ, 10; GK, 6; and HJ, 1. The resulting predicted distribution was then directly comparable to the global database data.
